# Accessibility of migrants to mental health services

**DOI:** 10.1192/j.eurpsy.2022.374

**Published:** 2022-09-01

**Authors:** A. Zartaloudi

**Affiliations:** University of West Attica, Nursing, Athens, Greece

**Keywords:** immigrants, accessibility, Mental health services

## Abstract

**Introduction:**

Cultural barriers and prejudices of mental healthcare professionals may promote inequalities in the provision of care to immigrant population and have a negative impact in provided service quality.

**Objectives:**

To identify barriers and facilitators of immigrants’ accessibility to mental health services.

**Methods:**

A literature review has been made through PubMed database.

**Results:**

Immigrants’ accessibility to mental health services may be related to social insurance problems, inadequate knowledge about their health rights, inadequate knowledge of the local language, as well as the bureaucracy of Greek State which may complicate mental health examination and treatment. The challenges faced by mental healthcare professionals in terms of diagnosis and treatment of migrants include communication difficulties due to linguistic and cultural differences as far as verbal presentation of symptoms and illness behavior is concerned. Culturally competent mental health professionals should work to erase racism and prejudice, to be familiar with cultural issues and have adequate knowledge related to cultural groups, to learn the life story of each patient separately and encourage patients to explain how their illness affects their lives, promoting a trustful communication environment in the context of healthcare provision.

**Conclusions:**

Exploring the specific needs of migrants as well as assessing the degree of satisfaction from their access to healthcare services are essential to providing integrated mental health care for people from different culture.

**Disclosure:**

No significant relationships.

